# Contribution of the *PALB2* c.2323C>T [p.Q775X] Founder mutation in well-defined breast and/or ovarian cancer families and unselected ovarian cancer cases of French Canadian descent

**DOI:** 10.1186/1471-2350-14-5

**Published:** 2013-01-09

**Authors:** Marc Tischkowitz, Nelly Sabbaghian, Nancy Hamel, Carly Pouchet, William D Foulkes, Anne-Marie Mes-Masson, Diane M Provencher, Patricia N Tonin

**Affiliations:** 1 Program in Cancer Genetics, Departments of Oncology and Human Genetics, McGill University, Montreal, Quebec, Canada; 2 Lady Davis Institute, Segal Cancer Centre, Jewish General Hospital, Montreal, Quebec, Canada; 3 Department of Medical Genetics, University of Cambridge, Cambridge, UK; 4 The Research Institute of the McGill University Health Centre, Montreal, Quebec, Canada; 5 Department of Medical Genetics, Jewish General Hospital, Montreal, Quebec, Canada; 6 Centre de recherche du Centre hospitalier de l′Université de Montréal (CRCHUM)/Institut du cancer de Montréal, Montreal, Canada; 7 Département de médicine, Université de Montréal, Montreal, Canada; 8 Division de gynécologie oncologique, et Département d’obstétrique et gynécologie, Université de Montréal, Montreal, Canada; 9 Department of Medicine, McGill University, Montreal, Quebec, Canada; 10 Department of Medical Genetics, Addenbrooke’s Treatment Centre, Addenbrooke's Hospital, University of Cambridge, Box 134, Level 6, Hills Road, Cambridge, CB2 0QQ, UK

**Keywords:** *PALB2*, p.Q775X, p.Q775*, Hereditary breast cancer, Breast cancer risk, Ovarian cancer, Founder mutations, French Canadians

## Abstract

**Background:**

The *PALB2* c.2323C>T [p.Q775X] mutation has been reported in at least three breast cancer families and breast cancer cases of French Canadian descent and this has been attributed to common ancestors. The number of mutation-positive cases reported varied based on criteria of ascertainment of index cases tested. Although inherited *PALB2* mutations are associated with increased risks of developing breast cancer, risk to ovarian cancer has not been fully explored in this demographically unique population.

**Methods:**

We screened the *PALB2* p.Q775X variant in 71 families with at least three cases of breast cancer (n=48) or breast and ovarian cancers (n=23) that have previously been found negative for at least the most common *BRCA1* and *BRCA2* mutations reported in the French Canadian population and in 491 women of French Canadian descent who had invasive ovarian cancer and/or low malignant potential tumors of the major histopathological subtypes.

**Results:**

We identified a *PALB2* p.Q775X carrier in a breast cancer family, who had invasive ductal breast carcinomas at 39 and 42 years of age. We also identified a *PALB2* p.Q775X carrier who had papillary serous ovarian cystadenocarcinoma at age 58 among the 238 serous subtype ovarian cancer cases investigated, who also had breast cancer at age 52.

**Conclusion:**

Our findings, taken together with previous reports, support adding *PALB2* c.2323C>T p.Q775X to the list of cancer susceptibility genes for which founder mutations have been identified in the French Canadian population.

## Background

Carriers of *PALB2* mutations in a heterozygous state have been associated with increasing the risk of developing breast cancer [1-10]. *PALB2* is a partner and localizer of the BRCA2 breast-ovarian cancer susceptibility protein to DNA damage sites [9,11]. Penetrance estimation for conferring breast cancer risk has been hampered by the paucity of cases, although estimates of 2- to 6-fold increased risk to breast cancer have been suggested [12,13], thus classifying *PALB2* as a moderate breast cancer risk allele [9,12-14]. Germline mutations in *PALB2* have also been identified in familial pancreatic cancer [15,16]. *PALB2* is comprised of 13 exons spanning a 38 kb region on chromosome 16p12.1 and mutation screening is complicated by the diversity of variants (including missense mutations) identified in cancer cases. The *PALB2* c.2323C>T mutation, which results in the introduction of a stop codon at amino acid position 775 (p.Q775X), has been reported in at least three French Canadian breast cancer families [5], and along with other protein truncating *PALB2* mutations found in breast cancer cases, is strongly suspected to be deleterious [17]. The French Canadian population of Quebec exhibits an unique genetic demography [18-20]. About 40% of French Canadian cancer families with at least three cases of breast and/ovarian cancer carry a pathogenic *BRCA1* or *BRCA2* mutation [20-25]. Although 15 different mutations in these genes have been reported in French Canadian cancer families, six specific mutations in *BRCA1* and *BRCA2* have been shown to account for a significant majority of mutation-positive families [20-26]. This has been attributed to a shared ancestry of mutation carriers due to common founders of the French Canadian population of Quebec [25-28].

The number of *PALB2* p.Q775X mutation-positive cases that have been reported thus far in studies involving the French Canadian population vary according to criteria and catchment area of ascertainment of index breast cases tested [5,29]. To further assess the contribution of *PALB2* p.Q775X mutation in the French Canadian population, we report the results of screening this variant in 71 well defined cancer families with at least three confirmed cases of breast and/ovarian cancer found negative for the most common *BRCA1* and *BRCA2* mutation reported in this population. We report the cancer phenotype of a new p.Q775X mutation-positive family. We also report the screening 385 invasive ovarian cancer cases and 106 low malignancy potential ovarian tumors not selected for family history of cancer that were ascertained from the French Canadian population, and describe the cancer history of the p.Q775X cases identified in this screen. We describe our findings in the context of previous studies describing mutation screens of *PALB2* in individuals of French Canadian descent.

## Methods

### Subjects and cancer families

The study subjects fall within two defined groups. The first group contains index cases from 71 independently ascertained families (Table 1). The index cases tested for mutations were recruited to the study through the hereditary cancer clinics in Montreal as part of research studies assessing the contribution of *BRCA1* and *BRCA2* in breast and/or ovarian cancer families as described previously [21,25]. They have a family history of breast cancer (n= 48) or breast and ovarian cancer (n=23) according to the following criteria: in addition to the index case affected with breast cancer at less than 66 years of age, the families contained at least two other confirmed cases of invasive breast and/or epithelial ovarian cancer in the same familial branch. The affected index cases from 26 breast cancer families (HBC) and 14 breast-ovarian cancer (HBOC) families were previously screened and found negative for BRCA1 and BRCA2 sequence variants by commercial DNA sequencing (Myriad Genetics, Myriad Genetics Laboratories, Salt Lake City, UT, USA). The index affected cases from the remaining 22 HBC and 9 HBOC families were found negative for 20 BRCA1 and BRCA2 mutations reported in French Canadian cancer families of Quebec which include the following most common *BRCA1* (c.4327C>T (R1443X), c.2834_2836delGTAinsC) and *BRCA2* (c.8537_8538delAG, c.5857G>T (E1953X), c.3167_3171delAAAAG) mutations reported in this population, as described previously [22,23,25]. All index cases in this study self-reported grandparental French Canadian ancestry. The second group contained 385 females with epithelial ovarian carcinomas and 106 low malignant potential tumors (Table 2), who were recruited to Banque de tissus et de données of the RRCancer of the Fonds recherché Québec-santé tumor bank between April 1991 and October 2007. At least 88% of all women with malignant serous, endometrioid or undifferentiated malignant ovarian cancer cases from RRCancer Tumor self reported French Canadian ancestry (unpublished data). All women with serous LMP (Low Malignant Potential) tumors self-reported FC ancestry [30]. None of these subjects were selected for family history of cancer. Histopathology according to criteria established by the International Federation of Gynecology and Obstetrics (FIGO), age at diagnosis and personal history of cancer were provided for each case. Written consent to participate was obtained and the study protocols approved by the ethics review boards of the University of Montreal Hospital Center, McGill University Health Centre and Jewish General Hospital.

**Table 1:** **Families and features of index cases screened for *PALB2 *c.2323C>T [p.Q775X] variant**

**Syndrome**	**Number of families**^**1**^	**Unilateral BC**	**Bilateral BC**	**BC and OC**	**OC**	**Mean age in years of BC (age range)**	**Mean age in years of OC (age range)**
HBC	48 [10]	45	3	0	0	46 (30–65)	n/a
HBOC	23 [4]	14	2	1	6	44 (25–55)	51 (31–74)
Total	71 [14]	59	5	1	6	46 (25–65)	51 (31–74)

^1^The numbers in the brackets refer to number of index cases where the complete coding region of *PALB2* genes was sequenced. Abbreviations: breast cancer (*BC*), hereditary breast cancer (*HBC*) hereditary breast and ovarian cancer (*HBOC*), and ovarian cancer (*OC*).

**Table 2:** **Features of ovarian tumors examined for *PALB2 *c.2323C>T [p.Q775X] mutation**

**Malignancy**	**Histology type**	**Number of cases**	**Prior history of BC**
Malignant	serous	238	10
Malignant	endometrioid	49	2
Malignant	mucinous	24	2
Malignant	clear cell	31	2
Malignant	undifferentiated	43	1
Low malignant potential	serous	56	4
Low malignant potential	endometrioid	2	0
Low malignant potential	mucinous	48	0
Total		491	21

Abbreviation: breast cancer (*BC*).

### *PALB2* mutation analysis

Mutation analysis was performed on DNA extracted from peripheral blood leukocytes or from fresh frozen tumor tissue. A sequence analysis of protein coding exons of *PALB2* for the index cases of 14 families was performed as described previously [5,10,17] (Table 1). The targeted analysis for *PALB2* c.2323C>T (p.Q775X) variant was performed using an allelic specific assay as described [5]. The variant-positive cases were confirmed by DNA sequencing using 3730XL DNA analyzer system platform from Applied Biosystems (Carlbad, CA, USA) at the McGill University and Genome Quebec Innovation Centre (Montreal, PQ, CDN). Sequences were compared with *PALB2* NCBI Reference Sequence *NM_024675* as described in GenBank (http://www.ncbi.nlm.nih.gov). For the Loss of heterozygosity (LOH) analysis, fresh frozen ovarian tumor tissue from the *PALB2* p.Q775X mutation-positive case was macrodissected and DNA was extracted from the collected cells using the QIAamp DNA Mini Kit (Qiagen). The PCR was carried out in a volume of 50 μL, as previously described [5,10]. Sequence data were analyzed using the Lasergene SeqMan Pro sequence analysis software by DNASTAR, Inc. (Madison, WI, USA) and Chromas 2.31 from Technelysium Pty Ltd. (Helensvale, Australia) and compared to the sequences from lymphocyte DNA from *PALB2* p.Q775X mutation-positive and mutation-negative cases.

The *PALB2* p.Q775X variant is also annotated as p.Q775* according to a recently proposed nomenclature alteration for nonsense changes by the Human Genome Variation Society (http://www.hgvs.org). However for historical purposes the p.Q775X designation is maintained in this report.

## Results

### *PALB2* c.2323C>T [p.Q775X] carriers in breast and/or ovarian cancer families

One *PALB2* p.Q775X positive case was identified among the cancer families not previously investigated for *PALB2* mutations. The index carrier case was identified among the total of 48 (2.1%) HBC families or 71 (1.4%) HBC and HBOC families that share a phenotype defined by at least three or more confirmed cases of breast and/or ovarian cancer in the same familial branch (Table 1). These families were previously found negative for *BRCA1* and *BRCA2* mutations or the most common pathogenic mutations in these genes found in the French Canadian population.

**Figure 1:**
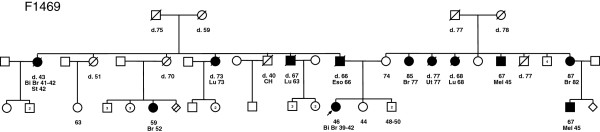
**Pedigree of *PALB2 *c.2323C>T [p.Q775X] mutation carrier family F1469.** An *arrow* indicates the proband and only known mutation carrier in family F1469. Abbreviations: bilateral breast cancer (*Bi Br*), cerebral hemorrhage (*CH*) esophageal cancer (*Eso*), lung cancer (*Lu*), melanoma (*Mel*), stomach cancer (*Sto*), and uterine cancer (*Ut*). Age at ascertainment and/or death (*d.*) are indicated if known along with ages at diagnosis of cancer.

The *PALB2* carrier had bilateral invasive ductal carcinomas of the breast at ages 39 and 42 and is part of the breast cancer family F1469 (Figure 1). Although breast cancer was reported in both paternal and maternal branches of her family (Figure 1), only the aunt and cousin from the paternal branch of the family were confirmed to have had breast cancer. Her paternal aunt also had bilateral invasive ductal carcinoma at ages 41 and 42, as well as atypical stomach carcinoma that was identified at age 42 but not further explored due to death soon thereafter. The carrier’s paternal cousin had an invasive breast cancer of mixed ductal and lobular histopathology at 52 and was still living at the time of pedigree analysis. Notable in this pedigree is that lack of ovarian cancer cases typified by *BRCA1* or *BRCA2* mutation carrier families. Her father had esophageal cancer and cancers of other sites reported (some confirmed) for both branches of her family. To our knowledge no other cases are available for genetic testing and thus transmission of the mutation is uncertain in this family. The family structure and associated phenotypes does not appear to overlap previously described *PALB2* p.Q775X positive families [5].

### *PALB2* c.2323C>T [p.Q775X] carriers in ovarian cancer cases

One *PALB2* p.Q775X positive case was identified among the 491 women with ovarian cancer or low malignant potential tumors. The carrier was diagnosed with a papillary serous cystadenocarcinoma at age 58. The carrier was identified among the 385 (0.3%) invasive ovarian carcinomas of all histopathological subtypes and among 238 (0.4%) invasive serous ovarian carcinomas (Table 2).

**Figure 2:**
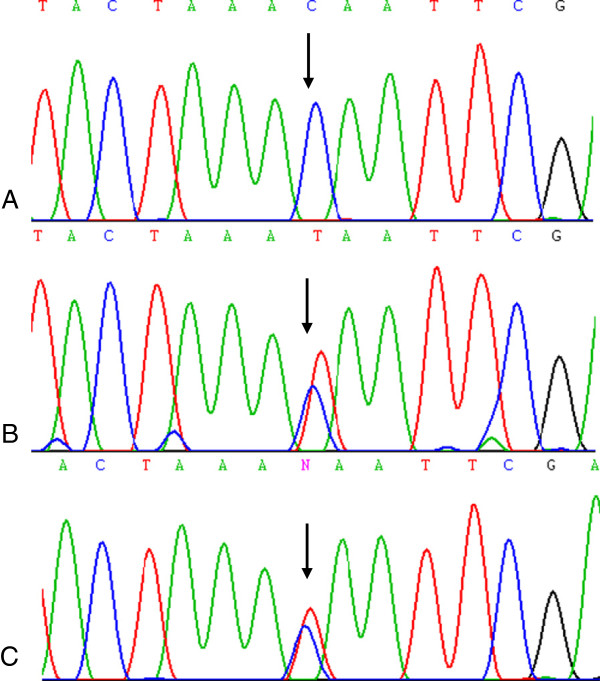
**Mutation analysis of *PALB2 *c.2323C>T [p.Q775X] containing region.** DNA sequencing chromatogram showing the region containing the c.2323C sequence of normal reference sample (*Panel ***A**) and corresponding interval from lymphocyte DNA of c.2323 T mutation carrier (*Panel ***B**) and ovarian cancer specimen DNA from the same patient (*Panel ***C**). The *arrow* indicates the position of the mutation in the sequence chromatogram.

There were 21 cases that also had a prior personal history of breast cancer and the *PALB2* p.Q775X carrier was in the group of 10 invasive serous ovarian carcinoma cases with this history (Table 2). The carrier had a breast cancer at age 52 years of undisclosed histological type. Genetic analysis of genomic DNA from ovarian cancer specimens did not indicate LOH of the *PALB2* locus (Figure 2) or identify a second mutation in the other coding exons of *PALB2*.

## Discussion

Our results are consistent with previously established frequency of *PALB2* c.2323C>T [p.Q775X] carriers in breast cancer families of French Canadian descent (Table 3). Initial reports of comprehensive screening of all of the protein encoding exons of *PALB2*, identified no variants in 38 breast cancer families, where 22 families had a prior probability of greater than 10% of harboring a *BRCA1* or *BRCA2* mutation [10]. The same group reported one of 50 (2%) breast cancer families, and two of 356 (0.6%) cases of early age (< 50 years) breast cancer with this mutation [5]. Pedigree analysis of the index *PALB2* p.Q775X positive cases from these three families indicated that they are not immediately related to each other and haplotype analysis was consistent with this being a founder mutation [5]. Four *PALB2* p.Q775X positive cases were also identified in a subsequent study involving 564 (0.7%) breast cancer cases not selected for family history of cancer, which also showed that 6% of cases harbored common *BRCA1, BRCA2* or *CHEK2* mutations [29]. This latter study also included the 356 early age breast cancer cases reported in a previous study, and one of these cases was found related to a member of previously reported mutation-positive family (P28031) and two other cases were the same carriers identified in a same previous study (P31030 and P26007) [5] (Table 3). In an independent study involving the investigation of a new *BRCA2* variant, c.9004 G>A (E3002K), a family (F1573) harboring both a *BRCA2* variant and the *PALB2* p.Q775X mutation was reported [22]. However, pedigree inspection revealed that family F1573 was related to one of three *PALB2* p.Q775X (P28031) families described in the initial report of this variant in the French Canadian breast cancer families [5]. The family history of *PALB2* p.Q775X carrier P36470 also identified in a screen of 564 breast cancer cases not selected for family history [29] appears not to be related to the *PALB2* p.Q775X carrier families described based on pedigree inspection, including family F1469 reported in this study. In summary, six *PALB2* p.Q775X breast cancer carriers which include five occurring in apparently unrelated cancer families have thus far been identified in screening breast cancer cases or breast cancer families.

**Table 3:** **Summary of *PALB2 *c. 2323C>T [p.Q775X] carriers identified in studies of French Canadian cancer families or cases**

**p.Q775X positive cases**	**Features of p.Q775X positive case [Family number]**	**Number of index cases screened**	**Context and feature of cases or families tested**	***BRCA1 *and *BRCA2 *status**	**Reference**
0		22	HBC and HBOC families; BRCAPRO scores > 0.10	Mutation-negative families	[10]
0		16	HBC and HBOC families; BRCAPRO scores < 0.10	Mutation-negative families	[10]
1	BC 54 [P28031^1^ and (F1573^2^)]	50	BC < 50 years of age, or BC between 50–65 yrs of age with at least one other BC or OC in first or second degree relative	Mutation-negative families	[5] ( [22])
2	BC 36 [P26007^1^] BC 49 [P31030^1^]	356^2^	BC <50 years	Common French Canadian mutation negative	[5]
4	BC 36 [P26007^1^] BC 46 [P28031^1^] BC 49 [P31030^1^] BC 49 [P36470]	564^2^	BC <50 years	Common French Canadian mutation negative	[29]
0		99	High risk BC families	Mutation negative	[31]
0		21	HBC and HBOC families with at least 2 first/second degree relatives with BC	Mutation negative	[2]
1	BiBC 39–42 [F1469]	48	HBC families (see Table 1)	Mutation-negative or common French Canadian mutation negative	This study
0		23	HBOC families (see Table 1)	Mutation-negative or common French Canadian mutation negative	This study
1	BC52;OC58	491	OC (see Table 2)	Not known	This study

^1^ Indicates cases identified in the pedigrees identified in independent studies involving 356 BC cases investigated in Foulkes et al. [5] overlap with series of 564 BC cases examined in a subsequent study reported by Ghadirian et al. [29]. ^2^Pedigrees P28031 and F1573 have related family members and were reported in independent studies [5,22]. Pedigree P28031 had two mutation-positives cancer cases, one as part of HBC family (BC 54) and the other recruited through BC<50 (BC 46) series. Abbreviations: breast cancer (*BC*), bilateral breast cancer (*BiBC*) ovarian cancer (*OC*) hereditary breast cancer (*HBC*), hereditary breast and ovarian cancer (*HBOC*).

In contrast, no *PALB2* variants were reported in two other independent studies involving 99 [31] and 21 [2] independently ascertained breast cancer families of French Canadian descent. Genealogy and genetic studies have reported variability of founder effects in various regions of Quebec [32], suggesting that demography may also be a factor in the paucity of *PALB2* p.Q775X carriers in some studies of French Canadian cancer families. The majority of our cases were ascertained in Montreal [20], whereas independent groups have ascertained their families from the Quebec City region [31]. This possibility could also account for the lack of *PALB2* p.Q775X carriers found in a screen of 6,440 newborns of French Canadian descent as the majority of these newborns were from the Quebec City region [5].

The young ages of breast cancer diagnoses and number of breast cancer cases per family in *PALB2* p.Q775X carrier families suggest that carriers of this mutation are at high risk for breast cancer (Table 3), as has been posited with some *PALB2* mutation carrier families [5,10,17]. Our findings here support this notion, as the *PALB2* p.Q775X carrier identified had a bilateral case of breast cancer diagnosed before age 45 years and a strong family history of breast cancer (Figure 1).

It is interesting that the *PALB2* p.Q775X carrier found among the ovarian cancer cases examined in this study had a prior history of breast cancer (Table 3). Notable is that her personal history of cancer does not match any of the cases that appear in the pedigrees of *PALB2* p.Q775X positive French Canadian cancer families described thus far (including the new carrier family identified in this study). The role of *PALB2* in ovarian cancer is uncertain, as there have been few documented ovarian carcinoma cases harboring germline mutations in this gene. Two *PALB2* mutation carriers were identified in a study of 339 unrelated ovarian cancer cases of Polish descent [3]. The carriers had high grade carcinomas of different histopathological types: serous (case diagnosed at 61 years) and endometrioid (case diagnosed at 54 years) subtypes, where the latter carrier also harbored a *BRCA2* mutation [3]. Two (0.6%) *PALB2* mutation carriers were reported in a study of 360 ovarian cancer cases that were also screened for *BRCA1, BRCA2* and other recently described cancer susceptibility genes [33]. Neither of these two high-grade serous carcinoma *PALB2* mutation-carriers (diagnosed at ages 51 and 58) had a personal history of breast cancer, although the ovarian cancer case diagnosed at age 58 years had a family history of breast and/or ovarian cancer [33]. A low frequency of *PALB2* carriers (0.4%) was also recently reported in an investigation of 253 ovarian cancer cases from the Volga-Ural region of Russia [34], with the only carrier identified in this study having a bilateral (moderate grade) serous ovarian carcinoma at age 46 and a prior history of melanoma. The low frequency of *PALB2* mutation carriers identified thus far may argue a minor role for this gene in conferring ovarian cancer risk compared with higher frequency of mutation carriers observed in breast cancer cases and breast cancer families. This is consistent with recent findings estimating that *PALB2* heterozygotes were 1.3-fold more likely to have a relative with ovarian cancer in the context of HBOC family history [2].

Our genetic analyses of the carrier ovarian cancer specimen harboring the *PALB2* p.Q775X mutation did not exhibit evidence of LOH of the *PALB2* locus. This could also be consistent with sufficient contamination of normal stromal DNA such that it would obscure an imbalance of alleles. It has been suggested that *PALB2* contributes to carcinogenesis through haploinsufficiency and/or a dominant negative effect given the paucity of LOH observed in the majority of breast cancer cases from *PALB2* carriers [3,4,6,10], with the exception of one study where a high frequency of LOH was seen [2]. LOH was observed for both ovarian cancer cases identified in one study [33]. Promoter methylation silencing has also been reported in four of 53 sporadic ovarian cancer cases [35]. The significance of these findings is unknown and warrants further investigation to elucidate the role of *PALB2* in both breast and ovarian carcinogenesis.

## Conclusion

The *PALB2* c.2323C>T [p. Q775X] mutation confers increased risk for breast cancer in the French Canadian population of Quebec. The contribution of *PALB2* c.2323C>T [p. Q775X] to the causation of breast cancer in French-Canadians appears to be lesser than that attributable to the most common founder alleles in *BRCA1* and *BRCA2*, but the young age at diagnoses and associated familial history of breast cancer suggest that this variant should be added to the panel of deleterious mutations screened for assessing breast cancer risk in this unique population. Indeed during the preparation of this manuscript another *PALB2* carrier harboring the p.Q775X variant was identified in the Hereditary Cancer Clinics affiliated with McGill University Health Centre. The carrier had bilateral breast cancer at ages 34 and 42 years and a strong family history of breast cancer further supporting the notion that *PALB2* p.Q775X carriers are at increased risk for breast cancer.

## Competing interest

The authors declared that they have no competing interest.

## Authors’ contributions

MT and PT conceived and oversaw the study and drafted the manuscript, NS and NH performed the molecular analysis. CP, WF, AM and DP recruited cases and provided clinical data. All authors read and approved the final manuscript.

## Pre-publication history

The pre-publication history for this paper can be accessed here:

http://www.biomedcentral.com/1471-2350/14/5/prepub
